# Reply to Dr. S. V. Clevenger

**Published:** 1882-01

**Authors:** A. D. Rockwell

**Affiliations:** 46 E. Thirty-first St.; New York


### ARTICLE IX.

EDITORS OF THE CHICAGO MEDICAL JOURNAL AND EXAMINER:—In an article by Dr. S. V. Clevenger, in the November number of your journal, I was more than surprised to see certain personal references. Whether in a scientific communication it is in good taste to descend to personal villification, may be safely left to the judgment of your readers, but certainly statements along that line that are untrue cannot be in any way defended. The former professional relationship between Dr. Beard and myself can be of but little interest to others; but as the author of the article in question has seen fit to go out of his way to assert that "the junior (?) partner of the firm became disgusted with the trickeries of the senior, and has since the dissolution of the partnership," etc., etc., I desire simply to say that the writer not only has no authority to make such statement, but that it is entirely without foundation in fact. Our partnership was amicably dissolved, and our relations are now friendly.

Allow me simply to refer to a letter by Dr. W. J. Morton, in the December issue of the JOURNAL AND EXAMINER, in which reference is made to the methods known as general faradization and central galvanization. He speaks of them as "pernicious and unscientific;" says that "protest against indiscriminate electrization should be entered again and again, until no reputable physician would dare to PUMP, so to speak, electricity into or through his patient," etc. Space cannot be allowed me here to show wherein these methods are both rational and scientific. This has been done elsewhere. This much may, however, be said, that, admitting the stimulating and tonic action of electrization, it can be no more unscientific to make the applications general, in those conditions where a general tonic action is indicated, than it is to submit the body to the influence of the shower bath, or the whole person to the action of the sunlight.

General faradization has, however, been used for so many years by numbers in the profession, with increasing gratification, that it seems strange at this date to read such an indiscriminate and wholesale denunciation as that indulged in by Dr. Morton. He might as justly object to anæsthesia, or in the use of Franklinic electricity, to treatment by insulation, which, although a good thing, might literally be termed a PUMPING IN. Among others, Dr. Vater, of the University of Prague, has, in a series of articles in the *Allgemeine Wiener Zeitung*, borne abundant testimony to the wide and varied therapeutic effects of general faradization.

Instead of subjecting the method to the test of theory alone, and then in his ignorance rejecting it, he has put it to the severer and more conclusive test of a patient and discriminating clinical investigation. Our work has been translated into German, and I may perhaps be acquitted of undue egotism when I say that it has been standard in Germany for years, and that the methods of general faradization and central galvanization therein described have been long used by some of her best men.

Rosenthal, especially, speaks enthusiastically of its value, and in this country, among others, Wier Mitchell understands and appreciatcs the benefits that follow its use.

As Dr. Morton, by his own admission (in answer to a question of mine at the last meeting of the American Neurological Association), has had absolutely no practical experience in the use of general faradization, some of the assertions in his communication to your journal can be safely characterized as somewhat premature and rash.

46 E. Thirty-first St., N. Y. A. D. ROCKWELL.

